# Supplementary material for: Conspecific and allospecific larval extracts entice mosquitoes to lay eggs and may be used in attract-and-kill control strategy
Source: Sci Rep. 2019 Sep 24;9:13747. doi: 10.1038/s41598-019-50274-1 (PMC6760493; doi:10.1038/s41598-019-50274-1)
Supplement: Supplementary file 1 — Supplementary Information [file 41598_2019_50274_MOESM1_ESM.pdf]

Supplementary Information

Conspecific and allospecific larval extracts entice mosquitoes to lay eggs and may be used in attract-and-kill control strategy

Gabriel B. Faierstein[1], WeiYu Lu[2], Andréa K.L.S. Sena[1], Rosângela M. R. Barbosa[1] & Walter S. Leal[2,*]

[1]Department of Entomology, Instituto Aggeu Magalhaes, Fundação Oswaldo Cruz, Recife-PE, 50740-465, Brazil

[2]Department of Molecular and Cellular Biology, University of California-Davis, Davis CA 95616 USA

*Corresponding author:

Walter S. Leal

wsleal@ucdavis.edu

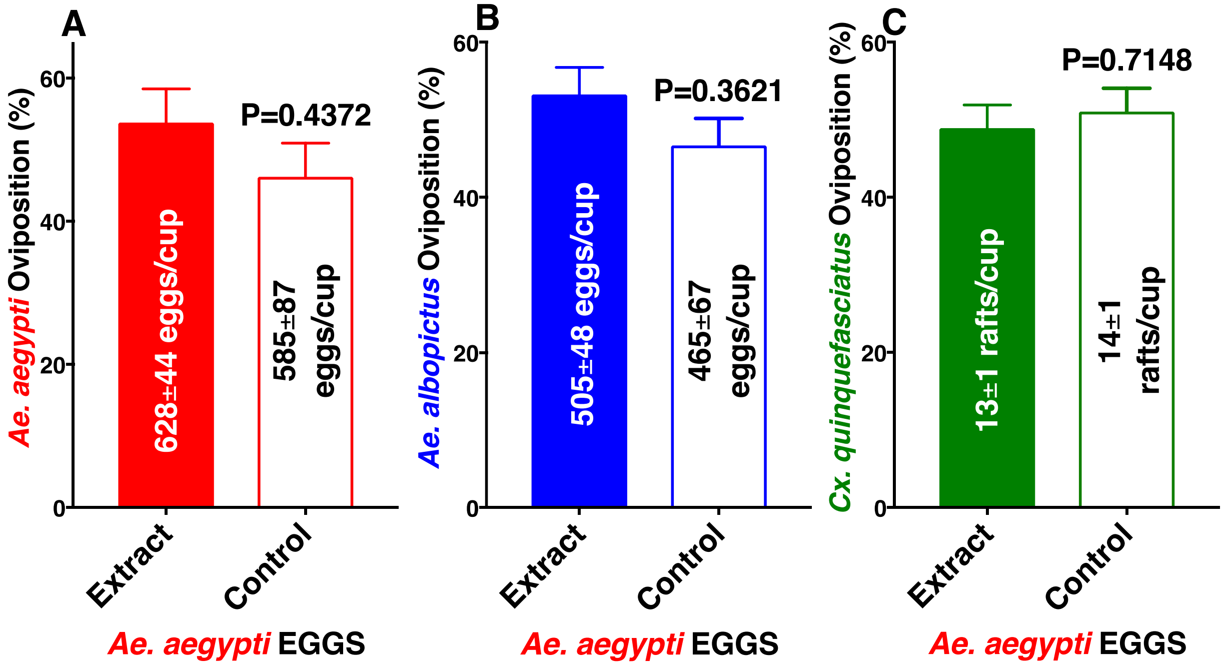

Figure S1. Oviposition preference by *Ae. aegypti*, *Ae. albopictus*, and *Cx. quinquefasciatus* to aqueous extracts from eggs of *Ae. aegypti* compared with water. Mean (±SEM) number of eggs laid by (A) *Ae. aegypti* and (B) *Ae. albopictus*, and egg rafts laid by (C) *Cx. quinquefasciatus* in cups loaded with extracts and control cups (water only). N = 10 for each treatment. For clarity, data are presented in percentage of oviposition preference, with mean number of eggs or egg rafts presented along with each bar. After arcsine transformation and passing the Shapiro-Wilk normality test, each dataset was compared by using the 2-tailed, paired *t* test.

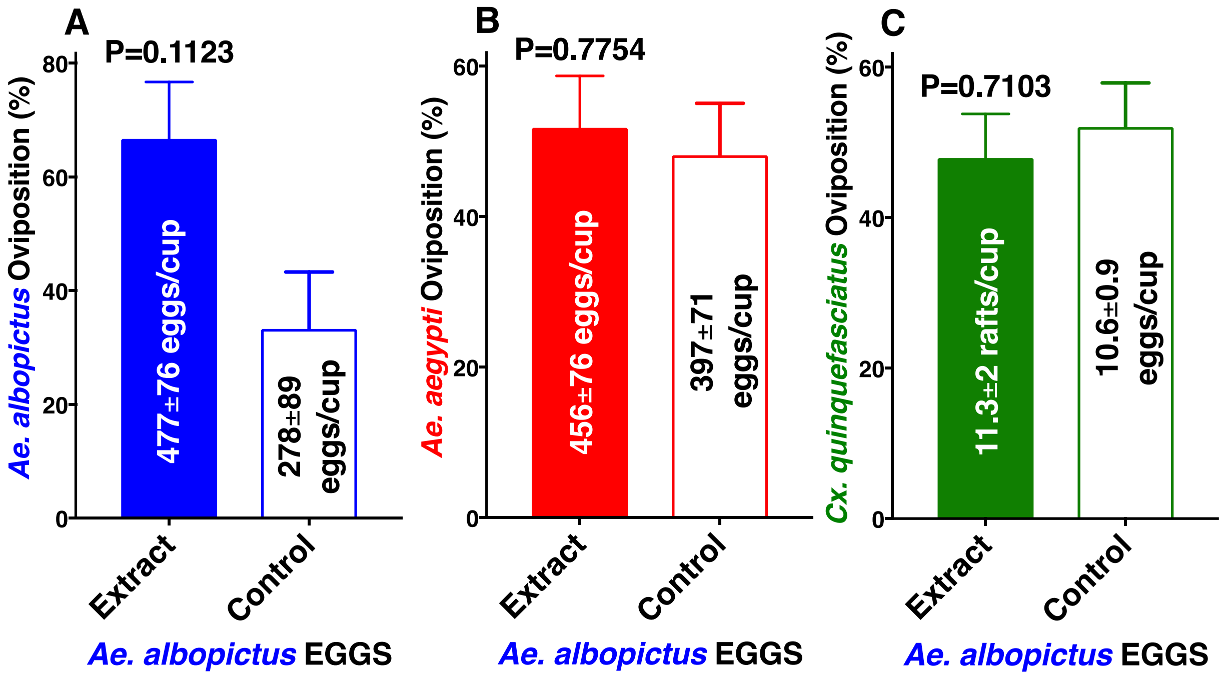

Figure S2. Oviposition preference by *Ae. albopictus, Ae. aegypti*, and *Cx. quinquefasciatus* to aqueous extracts from *Ae. albopictus* eggs compared with water. Mean (±SEM) number of eggs laid by (A) *Ae. albopictus* and (B) *Ae. aegypti*, and egg rafts laid by (C) *Cx. quinquefasciatus* in cups loaded with *Ae. albopictus* egg extracts and control cups (water only). (D, E, F) Oviposition preference by the same 3 species in dual choices assays comparing *Ae. albopictus* pupal extracts and water only. N = 10 for each treatment. For clarity, data are presented in percentage of oviposition preference, with mean number of eggs or egg rafts presented along with each bar. After arcsine transformation and passing the Shapiro-Wilk normality test, each dataset was compared by using the 2-tailed, paired *t* test.

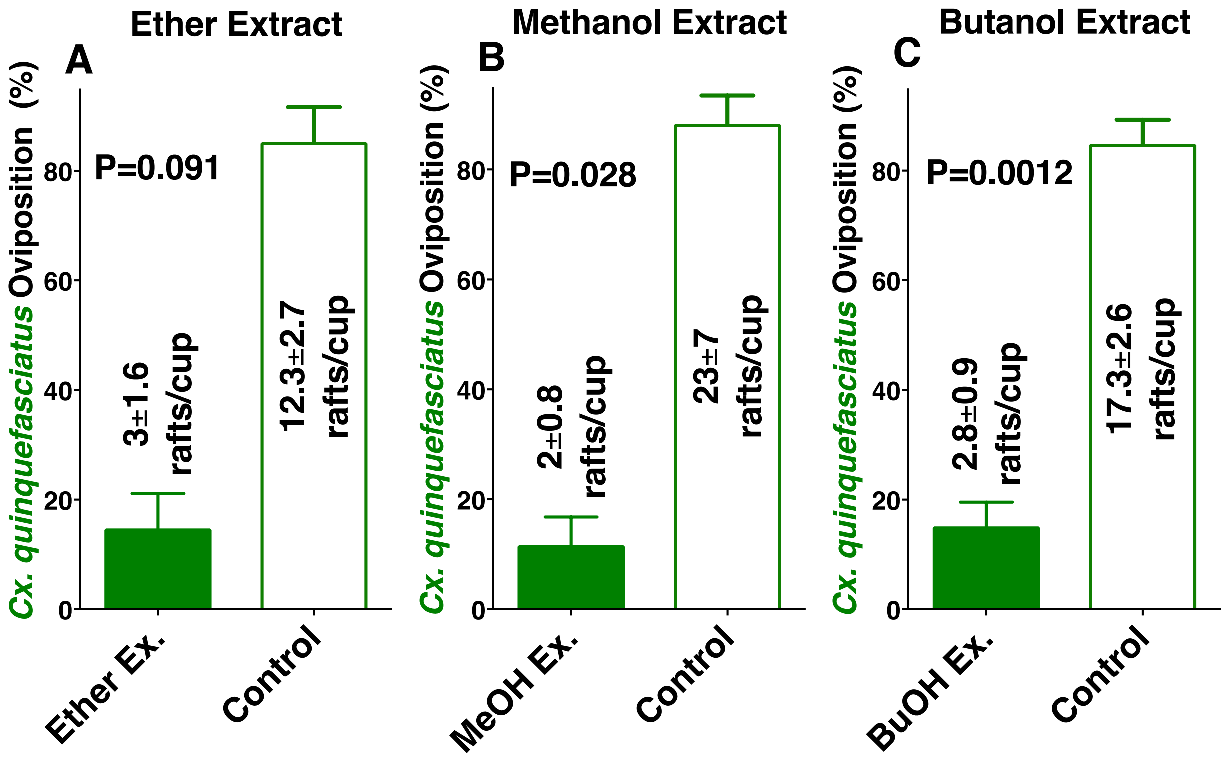

Figure S3. Oviposition preference by *Cx. quinquefasciatus* in dual choice assays comparing water vs conspecific larval extracts with various solvents. Percentage of oviposition preference comparing control traps with those loaded with (A) diethyl ether (ether), (B) methanol, and (C) butanol extracts from 4th-stage *Cx. quinquefasciatus* larvae. N = 12 for each treatment. After arcsine transformation and passing the Shapiro-Wilk normality test, each dataset was compared by using the 2-tailed, paired *t* test.

Raw Data for Figure 1

Ae. aegypti		Ae. albopictus		Cx. quinquefasciatus	
Extract	Control	Extract	Control	Extract	Control
419	32	791	223	18	9
1899	246	1266	306	8	14
1561	583	879	241	24	4
1549	54	1203	320	24	4
630	163	607	251	16	10
790	440	863	291	5	4
1417	309	721	329	13	9
1572	343	837	391	10	12
1916	793	921	408	18	10
504	291	1319	429	15	13

Raw Data for Figure 2

Ae. albopictus		Ae. aegypti		Cx. quinquefasciatus	
Extract	Control	Extract	Control	Extract	Control
878	551	850	558	22	3
1888	616	1287	92	10	4
498	155	807	563	23	2
231	183	1463	636	25	1
602	416	1357	1001	21	4
106	70	1623	133	19	4
475	45	843	369	17	4
509	94	518	262	25	3
1626	307	878	383	17	2
869	293	951	653	16	12

Raw Data for Figure 3

Cx. quinquefasciatus		Ae. aegypti		Ae. albopictus	
Extract	Control	Extract	Control	Extract	Control
2	1	302	90	331	274
15	1	814	20	796	147
28	2	1171	173	298	321
28	2	1524	230	199	40
2	0	1589	54	536	80
18	4	274	253	589	119
13	8	484	84	874	331
17	14	419	32	558	574
22	8	819	160	996	246
21	9	730	68	544	62

Raw Data for Figure 4

Ae. aegypti				Cx. quinquefasciatus			
Hexane Ex.	Control	DMSO Ex.	Control	Hexane Ex.	Control	DMSO Ex.	Control
1346	662	1221	421	18	25	24	34
929	847	1165	440	12	25	31	31
1329	1021	1067	235	14	15	34	8
1116	1094	998	430	15	46	37	14
1274	981	1093	294	34	16	38	22
874	1217	985	426	51	22	35	8
1120	1096	1139	262	3	59	49	20
1121	1094	1093	298	3	105	41	42
1206	983	1194	206	31	83	44	16
1047	871	846	397	29	63	44	14
1228	1374	981	235	25	18	21	10
1051	1197	1149	474	15	15	16	13

Raw Data for Figure 5

Aq. Phase	Control	Aq. Phase	Control
32	1	32	1
38	3	38	3
41	1	41	1
21	12	21	12

Raw Data for Figure 6

3 days				30 days			
4oC		Lyophilized		4oC		Lyophilized	
Extract	Control	Extract	Control	Extract	Control	Extract	Control
1286	299	1223	479	840	336	1094	289
1234	451	1662	156	657	362	893	380
1336	276	1266	532	872	793	953	314
1334	201	1240	174	553	542	1487	94
1454	363	1738	173	884	452	912	214
1502	414	1683	259	749	321	1188	384
1237	431	839	477				
550	327	906	357				
1384	373	1203	309				
969	424	1297	485				
684	207	817	406				
1208	329	659	198				

Raw Data for Figure 7

Control	0.1 L-eq/ml	0.33 L-eq/ml	0.66 L-eq/ml	1 L-eq/ml
26	187	414	571	584
12	78	485	679	711
79	188	122	556	270
105	105	357	492	928
174	132	280	511	930
19	42	543	89	847
100	282	340	660	208
73	128	131	178	547
192	433	131	493	712
0	357	699	406	480
129	591	235	551	772
67	81	434	404	332

Raw Data for Figure 8

Control+Bti	Extract+Bti		Control+Bti	Extract+Bti
22	1		80	110
73	2		29	59
72	176		40	146
1	90		125	206
37	129		0	57
43	58		57	71
127	291		0	127
585	443		156	193
28	51		48	103
45	59		153	232
0	120		42	136
130	157		197	95
0	47		102	122
44	231		9	47
256	682		92	203
167	610		75	173
36	60		217	262
3	4		33	50
274	466		130	299
115	237		217	416
46	161		192	441
130	157		31	111
0	47		150	389
44	231			
108	116			
97	115			
48	65			
5	201			

Raw Data for Figure S1

Ae. aegypti		Ae. albopictus		Cx. quinquefasciatus	
Extract	Control	Extract	Control	Extract	Control
637	484	234	181	9	12
723	634	561	484	13	15
568	963	527	634	17	12
831	245	596	663	12	10
354	857	697	245	8	22
697	755	587	669	15	14
586	478	496	755	13	16
468	214	633	478	14	12
713	896	466	214	18	13
698	325	258	325	15	14

Raw Data for Figure S2

Ae. albopictus		Ae. aegypti		Cx. quinquefasciatus	
Extract	Control	Extract	Control	Extract	Control
411	196	777	181	9	10
544	39	808	615	8	11
796	29	36	123	14	16
553	39	499	595	12	10
338	27	181	615	21	9
156	796	544	540	21	7
102	481	527	83	3	10
798	449	492	333	2	11
665	113	316	623	10	7
410	609	384	261	13	15

Raw Data for Figure S3

Ether Ex.	Control	MeOH Ex.	Control	BuOH Ex.	Control
1	6	0	17	2	25
5	13	1	21	1	25
2	23	2	9	2	17
0	5	2	4	3	11
10	16	6	46	2	12
0	11	1	41	7	14
